# Anthropometric and body composition characterisation of competition Portuguese crossfit athletes

**DOI:** 10.1080/07853890.2021.1896075

**Published:** 2021-09-28

**Authors:** João Oliveira, Luís Fonseca, Filipa Vicente, Paula Pereira

**Affiliations:** a Instituto Universitário Egas Moniz (IUEM), Egas Moniz Cooperativa de Ensino Superior, Caparica, Portugal; b Centro de Investigação Interdisciplinar Egas Moniz (CiiEM), Egas Moniz Cooperativa de Ensino Superior, Caparica, Portugal; c Grupo de Estudos em Nutrição Aplicada (GENA), Instituto Universitário Egas Moniz (IUEM), Egas Moniz Cooperativa de Ensino Superior, Monte de Caparica, Caparica, Portugal

## Abstract

**Introduction:**

Anthropometric characteristics and body composition are an important element in sports performance. Skinfold and circumference measurement had been recognised as an adequate and valid tool to assess body composition through specific equations from several authors which should be validated for each sport. The aim of this work was to characterise a Portuguese sample of competition athletes in Crossfit comparing several equations based in skinfold measurement.

**Materials and methods:**

This was an observational study conducted in a Crossfit box where 11 athletes were recruited, 7 were male and 4 female. The anthropometric assessment included the height measurement through a stadiometer (SECA123), a weigh in a digital scale with 100 g precision (ELECTRONIA) and the measurement of 7-site skinfold (bicep, tricep, subscapular, suprailiac, abdominal, thigh and calf) with a Slim Guide adipometer. Skinfold measurements were conducted according to ISAK protocol. All the results were treated with SPSS statistics 17.0 software.

**Results:**

The sample included 11 athletes where 4 were female (36%). Participants were 25–39 (31.6 ± 4.4) years old. The average body fat in male and female athletes was 13.7%±3.98 and 28.4%±5.6, respectively. These results were compared through a paired-samle *t*-test. In men, Durnnin & Womersley gave the highest body fat percentage while in female this and Jackson & Pollock where the highest, the lowest values were obtained through Evans formula in both genders. There were significant differences between all the formulas used for men (*p* < .05) but in women results only the formulas Durnnin & Womersley and Jackson & Pollock had shown significant differences (*p* < .05).

**Discussion and Conclusions:**

The sample of Crossfit athletes had shown values of body fat within the normal range. In spite of these, there were found some differences between several formulas which reinforces the need to use more than one formula in body fat assessment in nutrition clinical setting. Because this study was done in a convenience sample, the size and the heterogenous distribution of gender can be considered as study limitations.

